# Histopathological Spectrum of Gliomas and Its Immunohistochemical Correlation in a Tertiary Care Setup

**DOI:** 10.7759/cureus.65036

**Published:** 2024-07-21

**Authors:** Srishti Malla, Rupali Bavikar, Charusheela Gore, Ashish Chugh, Sushama Gurwale

**Affiliations:** 1 Pathology, Dr. D. Y. Patil Medical College, Hospital and Research Centre, Dr. D. Y. Patil Vidyapeeth, Pune, IND; 2 Neurosurgery, Dr. D. Y. Patil Medical College, Hospital and Research Centre, Dr. D. Y. Patil Vidyapeeth, Pune, IND

**Keywords:** ependymoma, astrocytoma, oligodendroglioma, isocitrate dehydrogenase, glioblastoma

## Abstract

Introduction

Central nervous system (CNS) tumors pose significant diagnostic challenges due to their varied morphological and differentiating characteristics. Modern advancements in immunohistochemistry (IHC) and molecular pathology have greatly enhanced prognostication, screening, and therapeutic management. Gliomas, a type of tumor originating from glial cells in the CNS, can develop from astrocytes, oligodendrocytes, or ependymal cells. According to the 2021 update, the classification of diffuse gliomas is primarily based on the presence or absence of isocitrate dehydrogenase (IDH1/2) mutations. IDH-wildtype gliomas (glioblastomas) have a significantly poorer prognosis compared to IDH-mutant gliomas (astrocytomas and oligodendrogliomas). Gliomas are highly infiltrative and resistant to treatment, making them largely incurable regardless of their grade and prognosis.

Objective

This study aimed to determine the histopathological diversity of gliomas and its correlation with protein expressions of IDH, ATRX gene (α-thalassemia/mental retardation syndrome X-linked), Ki-67, and p53 mutations (tumor suppressor gene-53), according to the 2021 World Health Organization (WHO) Classification of CNS Tumors, Fifth Edition.

Methods

This descriptive cross-sectional study was carried out in the Department of Pathology at a tertiary care center, focusing on various types of gliomas received over a two-year period. A total of 54 specimens of gliomas received from the Department of Neurosurgery were subjected to histopathological examination. Sections were stained using hematoxylin and eosin (H&E), and IHC was performed using four markers (IDH, ATRX, p53, Ki-67) in each case. Results were analyzed according to the 2021 WHO Classification of CNS Tumors, Fifth Edition.

Results

The majority of individuals were between the age group of 40 and 60 years, showing a male predominance (65%). The most common site was the frontal lobe. Glioblastoma constituted the largest proportion (46.2%) of the total cases, followed by astrocytoma (20.3%), oligodendroglioma (18.5%), pilocytic astrocytoma (7.4%), and ependymoma (7.4%). All 11 cases of astrocytoma exhibited IDH mutation and ATRX loss, with p53 positive in the majority of cases. Strong nuclear p53 immunohistochemical positivity in >10% of tumor nuclei correlates with TP53 mutations. Among 25 cases of glioblastoma, IDH was negative, ATRX was retained in all cases, and 11 cases were positive for p53 mutation. For oligodendroglioma, out of 10 cases, IDH mutation was positive, and ATRX was retained in all cases. p53 mutation was not seen in any case. All cases of pilocytic astrocytoma were negative for IDH and p53 mutations, with ATRX retained in all cases. In all cases of ependymoma, IDH and p53 mutations were negative, and ATRX was retained in all cases. Glioblastomas exhibited the highest Ki-67 expression.

Conclusion

The 2021 WHO Classification of CNS Tumors, Fifth Edition, was updated, building on previously established concepts and continuing to evolve. The final diagnosis of gliomas relies on a comprehensive combination of clinical evaluation, neuroimaging, pathological examination, and molecular analysis. Nonetheless, histopathological examination, along with IHC, remains the cornerstone of diagnosis.

## Introduction

Tumors affecting the central nervous system (CNS) constitute 1-2% of all neoplasms. CNS tumors present significant diagnostic challenges due to their diverse morphological and differentiating features, as well as varied histology. Nowadays, prognostication, screening, and therapeutic management of CNS tumors are greatly facilitated by advancements in immunohistochemistry (IHC) and molecular pathology [1].

Astrocytomas are tumors that arise from astrocytes, a type of glial cell in the CNS. Glioblastoma is the most aggressive and common type of primary brain tumor in adults. It originates from astrocytes and is highly infiltrative and resistant to treatment. Oligodendrogliomas are tumors that arise from oligodendrocytes, another type of glial cell, and have a better prognosis compared to astrocytoma and glioblastoma. Pilocytic astrocytomas are slow-growing tumors that commonly occur in children and young adults. They are typically well-circumscribed and often cured with surgical resection. They are considered grade 1 tumors. Ependymomas arise from cells lining the ventricles of the brain and spinal cord. They can occur at any age but are more common in children and young adults.

Approximately three-quarters of all primary brain tumors are gliomas, predominantly glioblastomas [2]. Gliomas are classified as grades 1-4 based on their aggressiveness or as astrocytomas, oligodendrogliomas, or ependymomas according to their cellular origin [3]. Ten percent of glioblastomas arise as secondary tumors from lower-grade gliomas, with the remaining 90% developing de novo [4]. Isocitrate dehydrogenase (IDH)-wildtype gliomas (gliomas that do not have mutations in the IDH genes), resembling glioblastomas, exhibit worse prognoses compared to diffuse IDH-mutated gliomas (IDH mutation confirmed on IHC), characteristic of lower-grade astrocytomas and oligodendrogliomas. IDH mutations frequently co-occur with p53 and ATRX mutations [5]. IDH mutation was analyzed via IHC, employing specific antibodies to identify and localize mutant IDH proteins in tissue samples. The antibodies used for IHC to detect IDH mutations include those targeting specific mutant IDH1 (R132H) proteins. In IHC, a mutation is defined by the presence of abnormal protein expression due to a genetic alteration. Positivity is defined when the antibody binds to the mutant protein, resulting in a visible staining signal in the tissue sample. The Ki-67 index is commonly used to assess cell proliferation [6].

The World Health Organization (WHO) introduced a new taxonomy and nomenclature for a broad spectrum of tumors, including gliomas, in its fifth edition of the CNS tumor classification, published in 2021 [7]. This updated classification integrates specific molecular alterations with tumor histology, aiming to enhance diagnostic precision [8].

The 2021 WHO Classification of CNS Tumors, Fifth Edition, has incorporated the "not elsewhere classifiable" (NEC) designation in addition to "not otherwise specified" (NOS). An NOS suffix in CNS5 indicates that the diagnostic information (histological or molecular) necessary to assign a specific WHO diagnosis is not available, providing an alert to the oncologist that a molecular work-up has not been undertaken or failed technically. An NEC suffix denotes that required diagnostic testing was successfully performed; however, discrepancies between clinical, histological, immunohistochemical, and genetic findings prevent a definitive WHO classification [9]. Gliomas are highly infiltrative and resistant to treatment, rendering them largely incurable irrespective of grade and prognosis. The presence or absence of IDH mutations primarily dictates the classification of diffuse gliomas under the 2021 guidelines [10].

Our study aims to assess protein expression of IDH, ATRX gene (α-thalassemia/mental retardation syndrome X-linked), Ki-67, and p53 mutations, alongside analyzing associated histopathological patterns.

## Materials and methods

This descriptive cross-sectional study was carried out in the Department of Pathology of Dr. D. Y. Patil Medical College, Hospital and Research Centre, Pune, India, focusing on various types of gliomas received over a two-year period from May 2022 to May 2024. Ethical approval was obtained from the Institutional Ethics Sub-Committee (IESC) of Dr. D. Y. Patil Medical College, Hospital and Research Centre under clearance number IESC/PGS/2022/197.

A total of 54 specimens of CNS space-occupying lesions received from the Department of Neurosurgery, diagnosed as gliomas, were included in our study and subjected to histopathological examination, irrespective of age and gender. Benign tumors and tumors not arising from the glial cell origin were excluded from the study. All submitted tissues were prepared by standard means and hematoxylin and eosin (H&E) stained for pathology evaluation. Reporting was done according to the 2021 WHO Classification of CNS Tumors, Fifth Edition.

IHC staining was done using one representative block in each case. It was performed by using a primary monoclonal antibody against the antigen for IDH, ATRX, p53, and Ki-67. All the procedures were carried out as per standard protocols. IHC for IDH, particularly IDH1 R132H, is used to detect mutations in the IDH1 gene. A positive result is indicated by the presence of diffuse brown staining in tumor cells. IHC for ATRX detects the presence or loss of ATRX protein. A positive result is indicated by the retention of ATRX staining in the nucleus of tumor cells. IHC for Ki-67 is used to assess the proliferation rate of tumor cells. We adopted a categorization approach based on groupings of five percentage points (e.g., 0-5%, 5-10%, etc.), given the absence of specific cutoff criteria. Strong nuclear p53 immunohistochemical positivity in >10% of tumor nuclei correlates with TP53 mutations in our study. The results were analyzed according to the 2021 WHO Classification of CNS Tumors, Fifth Edition.

The data were subsequently utilized to ascertain the relative frequencies of different histopathological patterns, along with the distribution of age, sex, and location of various CNS tumors. Data was collected using preformed data collection forms and case record forms. Standard statistical tests were applied to analyze the data. 

## Results

The study encompassed the analysis of a total of 54 glioma specimens. The majority of cases (50%) were in the age group of "40-60" years (n=27), followed by the age group of "20-40" years, accounting for 24% of the population (n=13). Those over 60 years old comprised 15% of the population (n=8), while those less than 20 years old exhibited the lowest incidence at 11% (n=6).

The study revealed a male predominance, with 65% (n=35). The most common space-occupying lesion was noted in the frontal lobe. The most prevalent symptoms observed were headache and limb weakness, followed closely by vomiting and seizures. Less frequently observed symptoms included ataxia and loss of memory.

In our study, the distribution of cases by tumor types showed that glioblastoma constituted the largest proportion, followed by astrocytoma, oligodendroglioma, pilocytic astrocytoma, and ependymoma (Table 1).

**Table 1:** Frequency of various gliomas The data has been represented as % CNS: central nervous system

CNS tumors (n=54)	Frequency (%)
Glioblastoma (n=25)	46.2
Astrocytoma (n=11)	20.3
Oligodendroglioma (n=10)	18.5
Pilocytic astrocytoma (n=4)	7.4
Supratentorial ependymoma (n=2)	3.7
Posterior fossa ependymoma (n=2)	3.7

Morphologically, the histology of individual cells of CNS WHO grade 4 IDH-mutant astrocytoma has considerable overlap with that of IDH-wildtype glioblastoma, and distinguishing between them requires testing for IDH mutations after H&E examination. Nevertheless, some features differ. Areas of palisading necrosis have been observed less frequently in CNS WHO grade 4 IDH-mutant astrocytomas than in IDH-wildtype glioblastoma. A typical glioblastoma on the H&E exam will be called grade 4 astrocytoma if IDH mutated and called glioblastoma if wildtype IDH according to the 2021 WHO Classification of CNS Tumors, Fifth Edition.

IHC was performed on each case in our study using the markers IDH, ATRX, p53, and Ki-67.

Out of 11 cases of astrocytoma, IDH mutation and loss of ATRX were noted in 100% of the cases. p53 mutation was found in the majority of cases (n=9), accounting for 81.8%. Around 18.1% (n=2) exhibited a negative p53 mutation.

Out of 25 cases of glioblastoma, IDH was negative (IDH-wildtype) and ATRX was retained in 100% of the cases. p53 mutation was present in 44% (n=11) and absent in 56% (n=14) of the cases.

For oligodendroglioma, out of 10 cases, IDH was positive and ATRX was retained in 100% of the cases. p53 mutation was not seen in any case.

There were a total of four cases of pilocytic astrocytoma. One hundred percent of the cases were negative for IDH mutation and p53 mutation. ATRX was retained in all cases.

A total of four cases of ependymoma were noted, out of which two were posterior fossa ependymoma and two were supratentorial ependymoma. In all four cases of ependymoma, IDH and p53 mutations were negative. ATRX was retained in all cases (100%) (Table 2).

**Table 2:** Distribution of mutations in various gliomas The data has been represented as % CNS: central nervous system; IDH: isocitrate dehydrogenase; p53: tumor protein 53; ATRX: alpha-thalassemia/mental retardation, X-linked

CNS tumors (n=54)	IDH mutation		p53 mutation		ATRX mutation	
	Positive	Negative	Positive	Negative	Loss	Retained
Astrocytoma (n=11)	11 (100%)	0 (0.0%)	9 (81.8%)	2 (18.1%)	11 (100%)	0 (0.0%)
Posterior fossa ependymoma (n=2)	0 (0.0%)	2 (100%)	0 (0.0%)	2 (100%)	0 (0.0%)	2 (100%)
Glioblastoma (n=25)	0 (0.0%)	25 (100%)	11 (44%)	14 (56%)	0 (0.0%)	25 (100%)
Oligodendroglioma (n=10)	10 (100%)	0 (0.0%)	0 (0.0%)	10 (100%)	0 (0.0%)	10 (100%)
Supratentorial ependymoma (n=2)	0 (0.0%)	2 (100%)	0 (0.0%)	2 (100%)	0 (0.0%)	2 (100%)
Pilocytic astrocytoma (n=4)	0 (0.0%)	4 (100%)	0 (0.0%)	4 (100%)	0 (0.0%)	4 (100%)

Table 3 shows the histopathological features of various glial tumors along with their median overall survival rate.

**Table 3:** Histopathological features of glial tumors with their median overall survival

Tumor type	Histopathological features	Median overall survival
Glioblastoma	Hypercellularity, necrosis, microvascular proliferation, mitosis	12-15 months
Astrocytoma	Usually hypercellular, nuclear atypia, fibrillary background	5-8 years
Oligodendroglioma	Uniform round nuclei, perinuclear halo, calcification	12-15 years
Pilocytic astrocytoma	Biphasic pattern, Rosenthal fibers, eosinophilic bodies	Long-term survival
Ependymoma	Perivascular pseudorosettes, true rosettes	5-10 years

**Figure 1:**
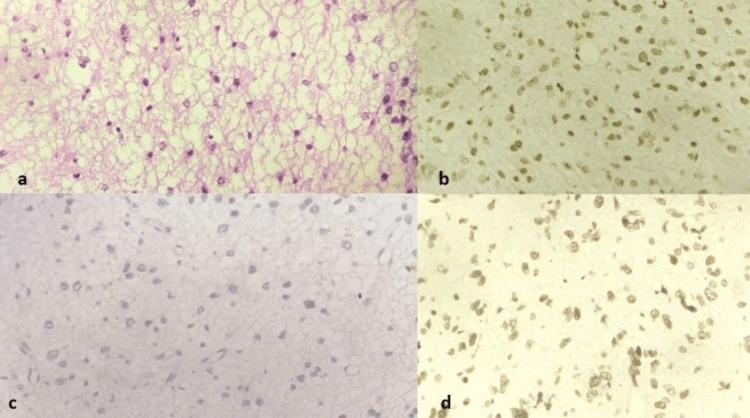
Astrocytoma showing (a) round to oval vesicular nuclei with indistinct nucleoli against a fibrillary background (H&E, 400×). (b) IDH is positive (H&E, 400×). (c) ATRX loss seen (H&E, 400×). (d) p53 shows positivity (H&E, 400×) H&E: hematoxylin and eosin; IDH: isocitrate dehydrogenase; ATRX: alpha-thalassemia/mental retardation, X-linked

Figure 1 shows the histopathological features of astrocytoma along with its IHC markers.

**Figure 2:**
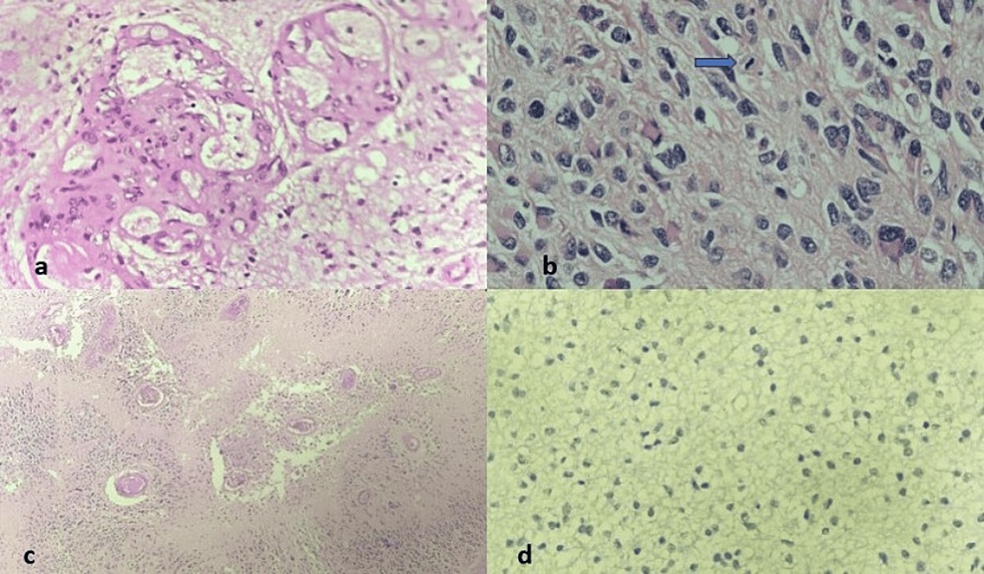
Glioblastoma showing (a) glomeruloid microvascular proliferation (H&E, 400×). (b) Mitosis is appreciated (H&E, 400×). (c) Pseudopalisading necrosis (H&E, 100×). (d) IDH is negative (H&E, 400×) H&E: hematoxylin and eosin; IDH: isocitrate dehydrogenase

Figure 2 shows the various features of glioblastoma along with its IHC markers.

**Figure 3:**
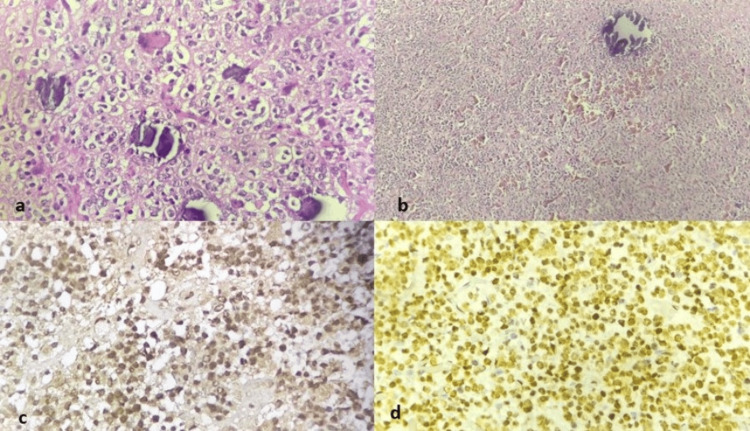
Oligodendroglioma showing (a) typical fried egg appearance (perinuclear clearing) with calcifications (H&E, 400×). (b) Chicken-wire vasculature (H&E, 100×). (c and d) IDH is positive and ATRX is retained, respectively (H&E, 400×) H&E: hematoxylin and eosin; IDH: isocitrate dehydrogenase; ATRX: alpha-thalassemia/mental retardation, X-linked

Figure 3 shows the features of oligodendroglioma along with its IHC markers.

**Figure 4:**
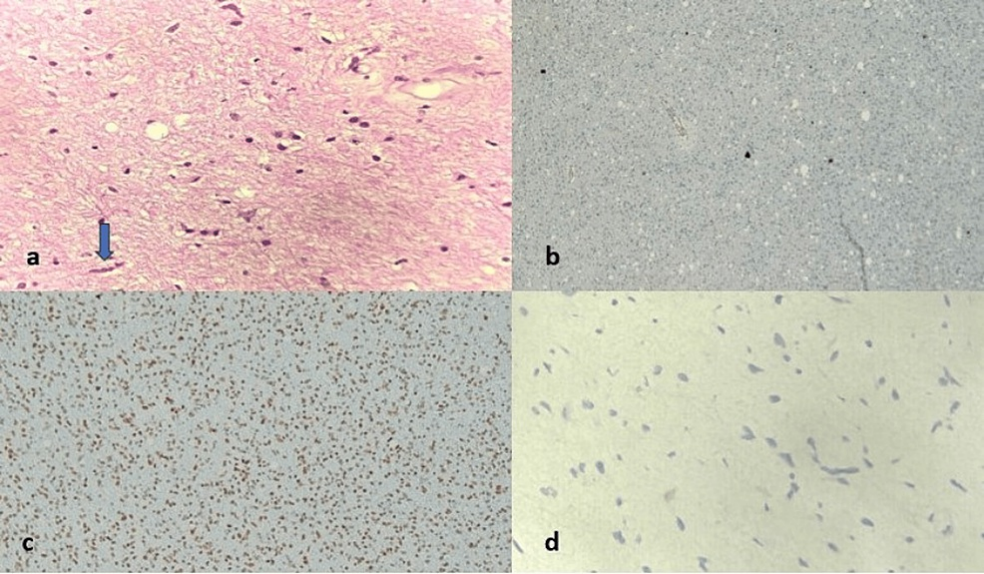
Pilocytic astrocytoma showing (a) Rosenthal fibers (H&E, 400×). (b) IDH is negative (H&E, 100×). (c) ATRX is retained (H&E, 100×). (d) Ki-67 proliferation <1% (H&E, 400×) H&E: hematoxylin and eosin; IDH: isocitrate dehydrogenase; ATRX: alpha-thalassemia/mental retardation, X-linked

Figure 4 shows the features of pilocytic astrocytoma along with its IHC markers.

**Figure 5:**
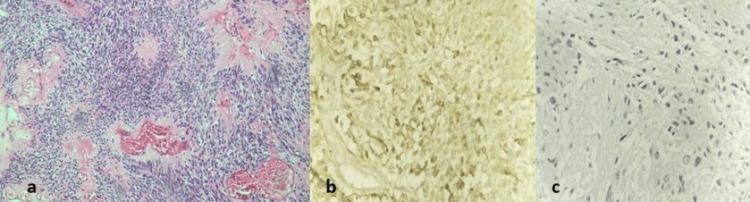
Ependymoma showing (a) perivascular pseudorosettes (H&E, 100×). (b and c) ATRX was retained and IDH was negative, respectively (H&E, 400×) H&E: hematoxylin and eosin; IDH: isocitrate dehydrogenase; ATRX: alpha-thalassemia/mental retardation, X-linked

Figure 5 shows the features of ependymoma along with its IHC markers.

Out of 11 cases of astrocytoma, 81.8% (n=9) demonstrated low Ki-67 levels of 0-5%, indicating a low level of proliferative activity. Nine percent (n=1) showed a Ki-67 level of 15-20%, and another 9% (n=1) showed a Ki-67 level of 20-25%.

Conversely, glioblastomas exhibited a high Ki-67 expression across the majority of cases, which reflects their aggressive nature. Twenty-eight percent (n=7) showed proliferation between 15% and 20%. Twenty percent (n=5) of the population of glioblastoma noted Ki-67 of 20-25%. Ki-67 of 25-30% was seen in 28% (n=7). Twenty-four percent (n=6) showed levels of Ki-67 more than 30%.

**Figure 6:**
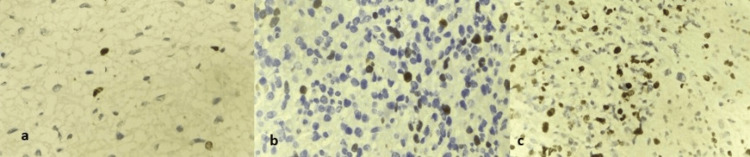
Ki-67 expression in (a) astrocytoma: 0-5% (H&E, 400×), (b) oligodendroglioma: 15-20% (H&E, 400×), and (c) glioblastoma: 25-30% (H&E, 400×) H&E: hematoxylin and eosin

All cases (100%) of pilocytic astrocytoma (n=4) and ependymoma (n=4) consistently exhibited low Ki-67 levels of 0-5%. In contrast, oligodendrogliomas displayed moderate to high Ki-67 expression, depending on the tumor grade. Forty percent (n=4) showed Ki-67 levels of 15-20%, 30% (n=3) showed Ki-67 levels of 20-25%, and 30% (n=3) of oligodendroglial tumors showed Ki-67 levels of 23-30% (Table 4). Figure 6 shows the Ki-67 expression of various gliomas.

**Table 4:** Ki-67 expression in different types of gliomas The data has been represented as % CNS: central nervous system

	Ki-67 expression (%)						
CNS tumors (n=54)	0-5	5-10	10-15	15-20	20-25	25-30	>30
Astrocytoma (n=11)	9 (81.8%)	0 (0.0%)	0 (0.0%)	1 (9.0%)	1 (9.0%)	0 (0.0%)	0 (0.0%)
Pilocytic astrocytoma (n=4)	4 (100%)	0 (0.0%)	0 (0.0%)	0 (0.0%)	0 (0.0%)	0 (0.0%)	0 (0.0%)
Oligodendroglioma (n=10)	0 (0.0%)	0 (0.0%)	0 (0.0%)	4 (40%)	3 (30%)	3 (30%)	0 (0.0%)
Glioblastoma (n=25)	0 (0.0%)	0 (0.0%)	0 (0.0%)	7 (28%)	5 (20%)	7 (28%)	6 (24%)
Ependymoma (n=4)	4 (100%)	0 (0.0%)	0 (0.0%)	0 (0.0%)	0 (0.0%)	0 (0.0%)	0 (0.0%)

## Discussion

This study aimed to examine the range of histopathological features of glial tumors and their immunohistochemical correlations within a tertiary care setting following the guidelines of the 2021 WHO Classification of CNS Tumors, Fifth Edition.

Recent advancements have made our nosology more accurate in the understanding of glioma pathogenesis and biology. The standard approach for assessing all diffuse astrocytic and oligodendroglial gliomas now begins with IHC to evaluate ATRX and IDH expression [11].

IDH mutations, which arise early in glioma development from stem cells capable of differentiating into both astrocytes and oligodendrocytes, are of particular interest [12]. The presence of IDH mutations in many glioma patients has sparked interest in understanding their roles in glioma progression [13].

The histology of gliomas is significantly influenced by IDH mutation status, which can be either positive (mutant) or negative (wildtype). Based on the IDH mutation status, additional IHC markers are used to identify various types of glial tumors.

Generally, for astrocytomas, IDH mutation is positive. The next marker to be applied is ATRX, which helps distinguish astrocytomas from oligodendrogliomas. A loss of ATRX confirms the diagnosis of astrocytoma. TP53 mutations vary with tumor grade. If ATRX is retained, the diagnosis is oligodendroglioma. Further grading is performed based on various molecular studies and histopathological characteristics, with p53 typically being negative. Histopathologically, grade 4 astrocytoma looks similar to grade 4 glioblastoma, but the IDH helps to confirm the diagnosis. According to the 2021 WHO Classification of CNS Tumors, Fifth Edition, if the IDH mutation is positive, it is termed astrocytoma, and if the IDH mutation is negative (IDH-wildtype), it is termed glioblastoma.

If IDH is wildtype (negative), the diagnosis is glioblastoma, where ATRX is usually retained. TP53 mutation can be either positive or negative. Ki-67 proliferation is usually greater in high-grade tumors.

Our study included a total of 54 glioma cases. Gender distribution among the study population revealed a male preponderance of 65%. A study conducted by Santosh et al. over a period of two years showed similar findings, with males (61%) being more affected than females. In the same study, diffuse gliomas were commonly found in the third and fourth decades [11]. Similar results were observed in a study by Sumathi et al. [14].

In our study, the majority of individuals fell within the age group of 40-60 years, making up 50% of the sample, which aligns with findings from the aforementioned studies.

The most prevalent clinical complaints seen in our study were headache and limb weakness, each reported in approximately 21% of cases, and the most common space-occupying lesion was located in the frontal lobe, accounting for 30% of cases.

Hamdani et al. reported findings similar to ours, noting the frontal lobe as the most frequently affected site and headache as the predominant symptom, followed by seizures [15]. Another study corroborated these results, also identifying the frontal lobe as the most common location for glial tumors (23.4%) and listing headache as the most common presenting complaint (62.3%), followed by seizures (28.9%) [11].

Dhar et al. and Ghanghoria et al. discovered that glioblastomas were the most prevalent type among all the tumors analyzed [16,17]. In another study conducted by Thota et al., they reported that out of 114 cases studied, glioblastomas accounted for 64.9% (74 cases), followed by anaplastic astrocytomas at 18.9% (22 cases) and diffuse astrocytomas at 16.2% (18 cases) [18]. In our study as well, the majority of cases were glioblastomas, comprising 46.2% of the total cases, followed by astrocytomas at 20.3%, oligodendrogliomas at 18.5%, pilocytic astrocytomas at 7.4%, and ependymomas at 7.4%.

IHC was performed on each case in our study. The markers used were IDH, ATRX, p53, and Ki-67. Out of 11 cases of astrocytoma, all exhibited IDH mutation and ATRX loss. p53 was positive in the majority of cases. Out of 25 cases of glioblastoma, IDH was negative, and ATRX was retained in all cases. Eleven cases were positive for p53 mutation, and 14 were negative. For oligodendroglioma, IDH was positive, and ATRX was retained in all cases. 1p19q co-deletion was not performed in our study due to cost constraints. All pilocytic astrocytomas were negative for IDH mutation and p53 mutation. ATRX was retained in these cases. For ependymoma, IDH and p53 mutation were negative. ATRX was retained in all cases. Glial fibrillary acidic protein (GFAP) was positive, and epithelial membrane antigen (EMA) showed perinuclear dot-like positivity in a few cases of ependymoma. GFAP was also positive in cases of astrocytoma.

In a study by Santosh et al., on pilocytic astrocytoma, it was observed that none of the cases showed IDH1, ATRX, or p53 mutations. Regarding WHO grade 2 tumors, which typically include diffuse gliomas of various types, the findings were as follows: IDH1 mutation was present in 10 out of 15 cases (66.7%), loss of ATRX was observed in 13 out of 15 cases (86.7%), and p53 mutation was detected in seven out of 15 cases (46.7%). These results highlight the prevalence of IDH1 mutations and ATRX loss in WHO grade 2 gliomas, consistent with their molecular features associated with lower-grade gliomas [11].

In the same study, focusing on anaplastic astrocytoma, WHO grade 3, the molecular characteristics were reported as follows: IDH1 mutation was present in two out of four cases (50%), ATRX loss was observed in three out of four cases (75%), and p53 mutation was detected in three out of four cases (75%). These findings indicate that while anaplastic astrocytomas generally exhibit more aggressive behavior compared to lower-grade tumors, they still demonstrate a variable but notable presence of IDH1 mutations, ATRX loss, and p53 mutations, which are common molecular alterations in astrocytic tumors. The molecular characteristics of WHO grade 4 tumors (glioblastomas) were reported as follows: IDH1 mutation was present in six out of 29 cases (20.7%), loss of ATRX expression was observed in 10 out of 29 cases (34.4%), and p53 mutation was detected in 25 out of 29 cases (86.2%). These results highlight the high prevalence of p53 mutation in glioblastomas, whereas IDH1 mutation and ATRX loss are less common in this aggressive tumor type. For oligodendrogliomas, among 10 cases of WHO grade 2 oligodendrogliomas, eight cases showed IDH1 mutation, and one case showed p53 mutation. Among 12 cases of WHO grade 3 anaplastic oligodendrogliomas, 10 cases showed IDH1 mutation. ATRX expression was retained in all cases of oligodendrogliomas. These findings underscore the characteristic molecular features of oligodendrogliomas, including frequent IDH1 mutation and retention of ATRX expression, which are consistent across different grades of oligodendrogliomas [11].

The same study also focused on glioblastomas categorized by age and IDH1 mutation status. For the >55-year age group, all 11 cases were IDH1 negative, indicating they were classified as glioblastoma, IDH1-wildtype. For the <55-year age group, out of 18 cases of glioblastoma, six cases were categorized as glioblastoma, IDH1 mutant type, due to immunoreactivity for IDH1, and 12 cases were categorized as glioblastoma, NOS (not otherwise specified), due to IDH1 negativity. These findings highlight the distribution of IDH1 mutation status in glioblastomas across different age groups, with a notable presence of IDH1 mutations in the younger age group and a higher proportion of IDH1-wildtype cases in the older age group [11].

Jiao et al. and Wiestler et al. reported that ATRX mutation occurs predominantly in diffuse astrocytomas and anaplastic astrocytomas but is very rare in primary glioblastomas (4-6%) and oligodendroglial tumors [19,20]. In another study referenced by Ebrahimi et al., it was found that ATRX expression was retained in all oligodendroglial tumors, ependymal tumors, and pilocytic astrocytomas [21]. Additionally, in a study focusing on grade 2 gliomas, none of the IDH-mutated, 1p/19q co-deleted tumors showed ATRX loss [22]. This suggests that ATRX loss is not a common feature in this specific subtype of diffuse gliomas. Recent studies have demonstrated a co-occurrence of ATRX loss and IDH mutations in a subset of diffuse gliomas, indicating a potential molecular relationship between these two markers in certain cases [23,24].

Regarding TP53 mutations, they are reported to be more prevalent in diffuse astrocytomas (74%), anaplastic astrocytomas (65%), and secondary glioblastomas (62%) compared to oligodendrogliomas (16%) or anaplastic oligodendrogliomas (9%) [12].

The Ki-67 index, which reflects cellular proliferation, is recognized as an independent prognostic factor in patients with glioblastomas. Some studies suggest that a higher Ki-67 index correlates with more aggressive tumor behavior and poorer prognosis in glioblastomas. This implies that tumors with a higher proliferation rate may be more resistant to treatment and associated with shorter survival times. Conversely, conflicting results in other studies indicate that the prognostic significance of the Ki-67 index may vary depending on factors such as treatment protocols, patient demographics (including age and gender), and tumor characteristics [25,26].

Arshad et al. noted that markers like Ki-67 and p53 tend to increase with the histologic grade of glial neoplasms. Several studies corroborate that histologic grade remains the most significant prognostic factor in gliomas. There is typically a strong correlation between increasing tumor grade, assessed by parameters like cellularity, nuclear atypia, mitotic activity, and features such as pseudopalisading necrosis or microvascular proliferation, and poorer prognosis. Higher grades of gliomas are generally associated with decreased survival times [27].

Dahlrot et al. present findings regarding the Ki-67 index in IDH1-wildtype glioblastomas. They observed that glioblastomas with an average Ki-67 index exceeding 20% predict a lower survival rate compared to those with lower Ki-67 index values. This suggests that higher cellular proliferation, as indicated by Ki-67, may correlate with poorer outcomes in this specific subtype of glioblastomas [28]. Similarly, another study found that Ki-67 levels significantly correlate with increasing grade of astrocytoma and patient age, but not with gender. This aligns with the understanding that Ki-67 is generally associated with more aggressive tumor behavior and higher grades in astrocytic tumors. Interestingly, in patients specifically diagnosed with glioblastomas, studies have reported no significant correlation between Ki-67 expression and overall survival. This discrepancy underscores the complexity of Ki-67 as a prognostic marker, as its significance may vary depending on tumor subtype, treatment regimen, and other clinical factors [28,29].

In our study, the majority of astrocytoma cases showed low Ki-67 levels, whereas glioblastomas exhibited high Ki-67 expression across the majority of cases. Pilocytic astrocytomas and ependymomas uniformly showed low Ki-67 levels. In contrast, oligodendrogliomas displayed moderate to high Ki-67 expression. The Ki-67 index in our study depended on the tumor grade. The higher the tumor grade, the higher the proliferation index of Ki-67.

Our study has a few limitations. The single-center design limits the generalizability of our results, and we could not conduct patient follow-up. Additionally, the sample size might be small, as many patients cannot afford IHC markers and molecular studies, which are essential for the precise diagnosis and targeted treatment of CNS tumors, despite the availability of multiple diagnostic modalities.

## Conclusions

IDH mutations are commonly found in diffuse lower-grade astrocytomas and oligodendrogliomas, and they are linked to a significantly better prognosis compared to diffuse IDH-wildtype gliomas, which often resemble IDH-wildtype glioblastomas. The term "diffuse glioma" is used when the tumor cells infiltrate the surrounding brain tissue extensively, making complete surgical resection challenging and often requiring additional treatment modalities such as radiation and chemotherapy. TP53, a tumor suppressor gene, is frequently mutated in most grade 2, 3, and 4 IDH-mutated astrocytomas. Although strong p53 protein staining indicates a TP53 mutation, it is less specific for diagnosing astrocytomas compared to the loss of ATRX expression. The level of Ki67 expression indicates cell proliferation and typically rises with higher tumor grades.

The 2021 WHO Classification of CNS Tumors, Fifth Edition, was updated, building on previously established concepts and continuing to evolve. The final diagnosis of gliomas relies on a comprehensive combination of clinical evaluation, neuroimaging, pathological examination, and molecular analysis. Nonetheless, histopathological examination, along with immunohistochemistry, remains the cornerstone of diagnosis.
